# The Histone H2A Variant MacroH2A1 Does Not Localize to the Centrosome

**DOI:** 10.1371/journal.pone.0017262

**Published:** 2011-02-22

**Authors:** Nathalie Friedman, Michal Barzily-Rokni, Sara Isaac, Amir Eden

**Affiliations:** Department of Cell and Developmental Biology, Institute of Life Sciences, The Hebrew University of Jerusalem, Jerusalem, Israel; University of Virginia, United States of America

## Abstract

MacroH2A1 is a histone H2A variant which contains a large non-histone C-terminal region of largely unknown function. Within this region is a macro domain which can bind ADP-ribose and related molecules. Most studies of macroH2A1 focus on the involvement of this variant in transcriptional repression. Studies in mouse embryos and in embryonic stem cells suggested that during early development macroH2A can be found at the centrosome. Centrosomal localization of macroH2A was later reported in somatic cells. Here we provide data showing that macroH2A1 does not localize to the centrosome and that the centrosomal signal observed with antibodies directed against the macroH2A1 non-histone region may be the result of antibody cross-reactivity.

## Introduction

MacroH2A1 is an unusual histone H2A variant. Its N-terminal domain is 64% identical to canonical histone H2A, while its C-terminal portion constitutes a large non-histone region (NHR) which is twice the size of the histone domain [1], [2]. Within the non-histone region is a protein domain known as the macro domain which was shown to bind ADP-ribose and related small molecules [3], but its function remains mostly unknown. In addition, the NHR has a less characterized linker with no known homology [4].

Most studies to date implicate macroH2A1 in regulation of gene expression and particularly in transcriptional repression. Examples include the recently described involvement of macroH2A in regulation of gene expression programs during cellular differentiation and development [5], [6], the transcriptional repression of HSP70 by recruitment of Parp1 to the promoter [7], the B-cell-specific repression of IL-8 [8], and the involvement of macroH2A1 in aberrant silencing of tumor suppressor genes in cancer [9].

Initially, however, most interest has focused on the enrichment of macroH2A on the inactive X chromosome (Xi) in female mammalian cells. Using immunofluorescent staining, it was demonstrated that macroH2A forms so called macro chromatin bodies (MCBs) representing focal macroH2A1 staining localizing to inactive but not active X [10], [11]. Formation of the MCBs was shown to be highly dependent upon XIST RNA. That is, removal of Xist in somatic female cells results in the disappearance of the MCB [12], while ectopic expression of Xist on autosomes results in the formation of ectopic MCB [13].

X-inactivation occurs during early embryo development. In pre-implantation female embryos, both X chromosomes are transcriptionally active. Immediately before gastrulation, either the maternally or the paternally derived X chromosome is inactivated in the embryo proper [14], [15]. The sequence of events during the process of X-inactivation can be analyzed in female embryonic stem cells which undergo X-inactivation once induced to differentiate [16]. Combining RNA fluorescence in situ hybridization (RNA-FISH) for detection of Xist RNA, with immunostaining against macroH2A1-NHR, showed that macroH2A enrichment at the Xi is a late event in the inactivation process suggesting macroH2A may be important for maintenance rather than establishment of the inactive state [13], [17]. A role for macroH2A1 in the silencing of Xi genes was later demonstrated [18],[19].

In undifferentiated ES cells (before X-inactivation), immunostaining with an antibody against macroH2A1-NHR further detected a densely stained region that did not co-localize with X chromosome(s) [20]. This structure was identified as the centrosome [21] and was also observed in early mouse embryos [22].

Time course analysis of macroH2A1 localization in differentiating female ES cells suggested that centrosomes of undifferentiated cells harbor a substantial store of macroH2A1 which is shuttled to chromatin and to the Xi upon differentiation. These observations suggested that macroH2A localization is developmentally regulated and suggested a role for the centrosome in the X inactivation process [21].

Later studies showed that the centrosomal association of macroH2A1 is not restricted to undifferentiated ES cells and is observed in both female and male somatic cells, both in interphase and in mitosis [22], [23].

Our attempt to understand the significance of macroH2A centrosomal localization resulted in several unexpected findings which lead us to conclude that macroH2A protein is not associated with the centrosome and that the centrosomal signal may be the result of antibody cross-reactivity.

## Results

### GFP-MacroH2A fusion protein does not localize to the centrosome

**Figure 1:**
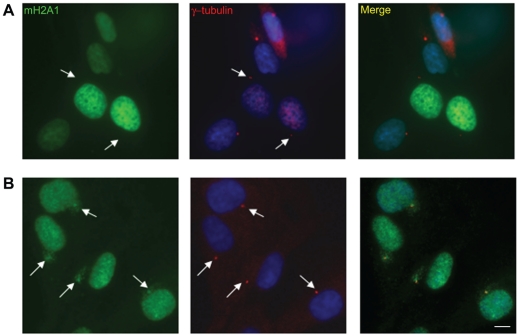
GFP fused macroH2A is not localized to the centrosome. **A**. WI-38 cells were transfected with GFP-macroH2A1 and immune-stained for γ-Tubulin as a marker of the centrosome (Red). **B**. WI-38 cells were co-stained for γ-Tubulin (red) and macroH2A1-NHR antibody (Green). DNA stained with DAPI (Blue). Bar indicates scale of 10 µm.

In an attempt to study the localization of macroH2A to the centrosome we generated a GFP fusion of macroH2A1. We observed localization of macroH2A1-GFP to chromatin and to the inactive X, in the form of macro-chromatin bodies (MCBs). However, we did not observe localization of GFP to the centrosome (Figure 1A). This was the case for all three macroH2A variants, in several cell types. Replacing GFP with RFP or moving the fusion from the C-terminus to N-terminus did not facilitate localization to the centrosome (data not shown). On the other hand, when the same cells containing tagged macroH2A1 where stained with the macroH2A1-NHR antibody, centrosomal staining was observed (Figure 1B).

### Centrosomal staining is evident in MacroH2A1 deficient cells

**Figure 2:**
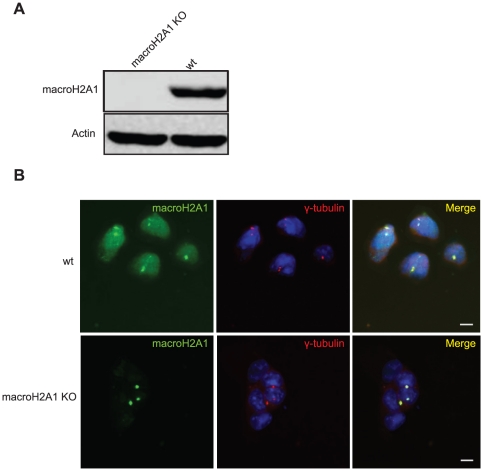
MacroH2A1-NHR antibody show centrosomal staining in macroH2A1 KO cells. **A**. Western blot of wt ES and macroH2A1 KO using the macroH2A1-NHR antibody confirms absence on macroH2A1 in knockout cells. **B**. Wt and macroH2A1 KO ES cells were stained with antibodies against γ-Tubulin (red) and macroH2A1-NHR (green). DNA stained with DAPI (Blue). Bar indicates scale of 10 µm.

In light of these results, we examined macroH2A1 localization in mouse embryonic stem cells (mESCs) in which both alleles of macroH2A1 are genetically targeted (Figure S1). Western blot analysis indicates complete loss of macroH2A1 at the protein level in these cells (Figure 2A). In immunofluorescence, wild type ES cells show nuclear staining as well as strong centrosomal staining. However, in macroH2A1 KO cells the nuclear macroH2A1 staining was lost while centrosomal staining was still evident (Figure 2B).

### Knock down of macroH2A1 removes nuclear but not centrosomal staining

The targeting approach used for generating macroH2A1 knockout mESCs removes only the second exon of the gene (containing the ATG) and obliterates the entire protein (Figure S1). However, the non histone region (NHR), which was used to develop antibodies against macroH2A1 is encoded by exons 5–10 [24]. It is therefore theoretically possible that the protein detected by the antibody is encoded by an alternative transcript which is not lost in the targeted cells and somehow not detected by western blot.

**Figure 3:**
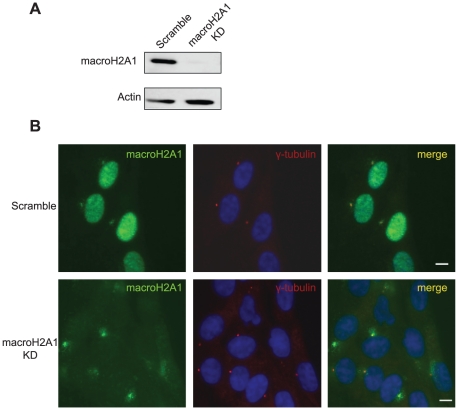
MacroH2A1-NHR antibody shows centrosomal staining in macroH2A1 KD cells. **A**. Western blot verifying KD efficiency. **B**. WI-38 were transduced with either a scrambled vector or a macroH2A1 KD Cells were then subjected to immunofluorescence using antibodies against γ-Tubulin (red) and macroH2A1-NHR (green). DNA stained with DAPI (Blue). Bar indicates scale of 10 µm.

To test this possibility, we used a lentiviral vector carrying shRNA to stably knockdown macroH2A1 (Figure 3). This shRNA targets a sequence within the NHR and should eliminate all transcripts running through this region. As before, nuclear staining was significantly reduced as a result of the knockdown, while centrosomal staining was unaffected even several weeks after the shRNA transduction and selection (Figure 3 and Figure S2a).

### Concurrent abolishment of macroH2A1 and macroH2A2 leaves centrosomal staining intact

**Figure 4:**
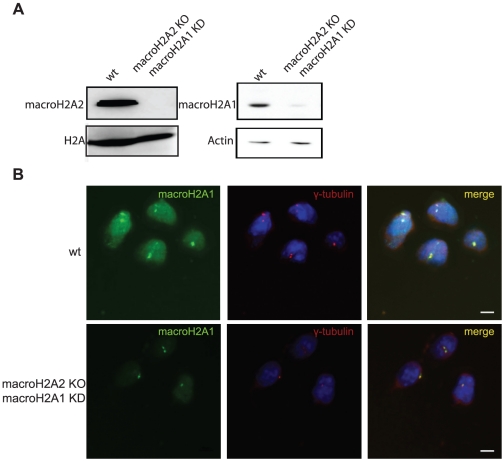
MacroH2A1-NHR antibody shows centrosomal staining in macroH2A1 and 2 double deficient cells. To generate double deficient cells macroH2A1 was knocked down in macroH2A2 KO mESCs using stable shRNA lentiviral transduction **A**. Western blot analysis confirming absence of macroH2A2, using affinity purified macroH2A2 antibody (left panel), and macroH2A1, using macroH2A1-NHR (right Panel) in the targeted cells compared to control. **B**. Wt ESCs and macroH2A1 and 2 double deficient cells were stained with antibodies against γ-tubulin (red) and macroH2A1-NHR (green). Nuclei were stained with DAPI (Blue). Bar indicates scale of 10 µm.

We considered the possibility that the antibody developed against macroH2A1 could cross react with the macro domain of macroH2A2 and that macroH2A2 was responsible for the centrosomal staining (although the macroH2A1-NHR antibody does not recognize macroH2A2 in western blots). Using macroH2A2 knockout ES cells (Figure 4A), and even macroH2A2 knockout ES with knockdown of macroH2A1, the centrosomal staining was still apparent indicating that none of the three macroH2A variants is responsible for the centrosomal signal (Figure 4B). Here again, quantification of centrosomal staining intensity indicated no significant difference between control and KO/KD cells (Figure S2b).

### Affinity purified macroH2A isoform specific antibodies do not stain the centrosome

**Figure 5:**
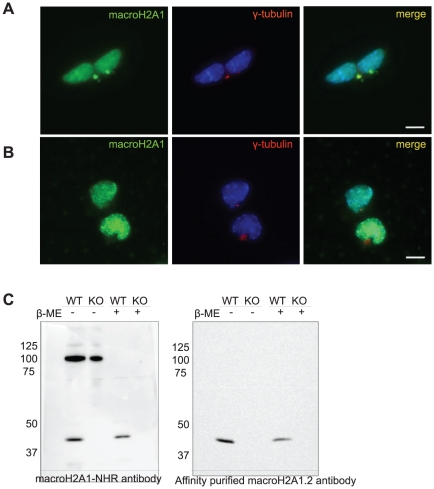
Affinity purified variant specific macroH2A1.2 antibody show no centrosomal staining. Wt ES cells were stained with antibodies against either (**A**) the macroH2A1-NHR antibody (green) or (**B**) with the affinity purified variant specific macroH2A1.2 together with γ-Tubulin (red). Nuclei were stained with DAPI (Blue) **C**. Protein samples were collected from wt ES and macroH2A1 KO ES in SDS containing sample buffer with or without β-mercaptoethanol. Western Blot analysis was done using anti-MacroH2A1-NHR or anti-MacroH2A1.2 isoform specific affinity purified antibody.

Studies that report centrosomal localization of mH2A1 [21], [22] used an antibody described in [10] [25] which was developed against the large non-histone region of macroH2A1.2. Importantly, when we used an isoform specific antibody which was developed using a specific peptide and was affinity purified using this peptide (mH2A1.2 amino acids 198–228) [26] only nuclear staining was detected (Figure 5).

### Western blot analysis using non-denaturing gel reveals additional band when using macroH2A1-NHR antibody

We suspected that the centrosomal signal may be caused by cross reactivity of the antibody with a different protein which is detected by the antibody on fixed cell samples but not following denaturation and western blot. We therefore checked if performing western analysis under less denatured conditions might facilitate the identification of an additional protein. Indeed when β-mercaptoethanol was omitted from the sample buffer, an additional band at the size of ∼100 kDa was observed. This band was not observed in samples containing β-mercaptoethanol (Figure 5C). Importantly, this second band observed using non-reducing conditions remained when investigating macroH2A1 knock-out samples (Figure 5C), indicating that it cannot be a multimeric version of macroH2A1. Moreover, exposing the same gel to the affinity purified variant specific antibody obliterated this band also in the non-denaturing conditions (Figure 5C). Thus, the presence of this band in western analysis is in full agreement with the presence of centrosomal staining, suggesting that the additional band might be the source of the centrosomal macroH2A1 artifact.

## Discussion

We provide several lines of evidence which are inconsistent with centrosomal localization of macroH2A1: First, we find that GFP-tagged macroH2A variants show no centrosomal signal (Figure 1 and data not shown). This is in agreement with other studies that used FLAG tagged macroH2A and also fail to observe macroH2A at the centrosome in somatic cells and in mouse oocytes early embryos [23], [27]. In addition, when using the macroH2A1-NHR antibody for immunofluorescence in macroH2A1 deficient cells, whether macroH2A1 KO or KD, the centrosomal signal remains (Figures 2 and 3). The centrosomal signal also persists when cells are deficient in both the macroH2A1 and macroH2A2 variants (Figure 4). We further provide evidence indicating that under more native conditions macroH2A1-NHR antibody can cross react with a different protein with apparent molecular weight of ∼100kDa (Figure 5C), suggesting that the centrosomal staining which is characteristic of this antibody may be the result of antibody cross-reactivity.

The initial reports detecting macroH2A at the centrosome used a polyclonal antibody raised against the whole NHR of macroH2A1.2 [10]. Later reports, including the current study, used an independent preparation of the antibody raised against the same epitope [25] and show the same apparent cross reactivity. In contrast, when a small portion of the protein was used to raise an antibody [26], it failed to identify the centrosome (Figure 5A and B). In itself, antibody cross reactivity is not unusual. However the possibility that it occurred with two independent antibody preparations and is apparent only under more native conditions suggests that there is a spatially similar structure on a different protein which is found at the centrosome. One reasonable possibility is that the antibody cross-reacts with a macro domain of another protein. There are at least 28 additional human proteins with a macro domain. None of them was reported to be found at the centrosome, but our results do not exclude this possibility.

In addition to immunofluorescence, both [21], [22] provide biochemical support to the presence of macroH2A1 in the centrosome by showing macroH2A in preparations of centrosomes. While normally this can be considered strong experimental confirmation, both studies used a protocol based on Mitchison and Kirschner [28], [29]. Importantly, in their protocol, Mitchison and Kirschner specifically mention that in interphase cells the centrosome is tightly attached to the nucleus and discuss at length the difficulties in separating the two. It is therefore possible that macroH2A detected in centrosome preparations was the result of contamination by nuclear material.

Chadwick et al propose that macroH2A may be shuttled to the centrosome for proteasomal degradation [23]. In our analysis we find the centrosomal signal even in macroH2A1 knockout or knockdown cells which are completely devoid of the nuclear protein (Figures 2 and 3). These results suggest that the origin of the centrosomal protein cannot be the nuclear full length macroH2A.

To conclude, while we do not identify the component responsible for the centrosomal signal observed with macroH2A1-NHR antibody, we provide strong evidence this signal does not represent macroH2A1.

## Materials and Methods

### Cell culture

Mouse ES cell line V6.5 [30] were grown without feeders in DMEM supplemented with 10% fetal calf serum (FCS), penicillin (50 mg/ml), streptomycin (50 mg/ml) and 2 mM (L-Glutamine), 0.1 nM non essential amino acids (NEAA), 0.1 mM β-Mercaptoethanol and 2×10^3^units/ml Leukocyte Inhibitory Factor (LIF). HEK293T [31] cells were cultured in DMEM medium and Tert-immortalized WI-38 cells ([32], ATCC number CCL-75) in MEM Both media were supplemented with 10% fetal calf serum (FCS), penicillin (50 mg/ml), streptomycin (50 mg/ml) and 2 mM (L-Glutamine). In addition, WI-38 medium contained 0.1 nM NEAA and 1 mM sodium pyruvate.

### Antibodies and Western blot analysis

Anti-macroH2A1-NHR polyclonal antibody is described in [25] and was prepared against the entire non-histone-region as in [10]. Affinity purified isoform specific Anti-macroH2A1.2 and Anti-MacroH2A2 were kindly provided by A. Ladurner (EMBL) [26]; Anti-β-actin (Abcam ab6276); Anti-H2A (Upstate 07-146). Anti-γ-tubulin (Sigma) Secondary antibodies coupled to horseradish peroxidase, FITC or Cy3 (Jackson Immunoresearch Laboratories).

### shRNA lentiviral vectors

Construction of lentiviral vectors pLKO1.puro/hygro carrying shRNA directed against mouse macroH2A1 was as described [31]. The macroH2A1 target sequence used was 5′-CCAGTTACTTCGTGTCTACAA-3′. Scrambled sequence was 5′-GTTGCCCCTAGACTTAGAACT-3′. Viral particles were produced in 293T cells as described in Moffat et al [31] and target cells were exposed to two rounds of infection followed by the relevant selection (2 µg/ml puromycin or 140 µg/ml hygromycin) for approximately 10 days. Knockdown cells used for the experiments presented in this study were analyzed weeks after the completion of selection.

### Immunofluorescence: and image analysis

Cells were plated on glass slides the day before the stain, fixed in 4% paraformaldehyde (PFA) for 10 min, and permeabilized for 5 min in 0.2% Triton X-100/PBS. Slides were then incubated in PBS containing 0.2% Tween 20, blocked with 10% fetal bovine serum and incubated with primary antibody for 1 h at room temperature. After three consecutive 5 min washes in 0.2% Tween 20/PBS, cells were incubated for 1 h with secondary antibody and DAPI followed by three additional washes before mounting [19]. Images were collected on a Nikon TE-2000 inverted microscope and processed using NIS-element software. Identical camera and microscope settings were employed to allow valid comparison between images of control and macroH2A1 deficient cell populations. In some images the red channel was enhanced to specifically point out the centrosomal signal of γ-tubulin. Quantification of the Intensity of centrosomal staining on the green channel (macroH2A1-NHR antibody stains) was performed on the original exposure using the Image Gauge v 4.0 software. The centrosomal signal was defined by summing the intensity from an area representing the centrosome and subtracting the background intensity of an identical area. For each centrosome a background signal corresponding to the centrosomal localization was used (nuclear or cytoplasmic).

### Generation of GFP-tagged macroH2A and transfection

Full-length mouse macroH2A variants tagged with enhanced green fluorescent protein (EGFP) at the C- and N-termini were generated by cloning PCR amplified cDNA of macroH2A into the EcoRI sites of pEGFP-C1 and pEGFP-N1 cloning vectors (Clontech) in frame, followed by sequencing of the plasmid insert. Transfection of the EGFP-macroH2A1 vectors was performed using the TransIT®-LT1 transfection agent (Mirus) according to the manufacturer's protocol.

## Supporting Information

Figure S1 **Generation of macroH2A1 V6.5 ES KO cells.** **A.** The second exon of macroH2A1 contains the translation initiation codon. The next in-frame ATG is close to the end of the protein and several out-of–frame sites are found between the two. Thus, removal of exon 2 should completely prevent production of the protein. The targeting vector included a genomic fragment that covers exon 2 with flanking regions. LoxP sites (blue triangles) and the Hygro-TK (HTK) selection marker were inserted as shown. After transient exposure to Cre recombinase and selection with Ganciclovir two versions were obtained: in one version exon 2 was removed producing a null allele (N), in the second version only the HTK cassette was lost producing a conditional allele that can be looped out in a later stage to produce a null allele. On the left pane: Southern blot analysis (using a probe from macroH2A1 exon 3) demonstrating the correct targeting of ES cells carrying a targeted allele (T/+) a conditional allele (C/+) or a null allele (N/+). In C/+ cells the targeting procedure was repeated and after transient exposure to Cre recombinase, N/N cells were obtained. **B.** Western blot using the macroH2A1-NHR antibody confirms that knockout (N/N) cells do not express macroH2A1 in. Coomassie stain is used as loading control.
(TIF)

Figure S2 **MacroH2A knockout or knockdown does not affect centrosomal staining intensity. Intensity of centrosomal staining was measured on the green channel using the Image Gauge v 4.0.** The centrosomal signal was defined by summing the intensity from an area representing the centrosome and subtracting the background intensity of an identical area. For each centrosome a background signal corresponding to the centrosomal localization was used (nuclear or cytoplasmic). Graph shows average intensity of centrosomal signal (arbitrary units +/-SD, N = 10) in A: cells transduced with either control or macroH2A1 shRNA (corresponding to Figure 3). B: wt mESCs compared to macroH2A2 KO cells transduced with macroH2A1 shRNA (corresponding to Figure 4).
(EPS)
