# Supplementary figures and images for: The Histone H2A Variant MacroH2A1 Does Not Localize to the Centrosome
Source: PLoS One. 2011 Feb 22;6(2):e17262. doi: 10.1371/journal.pone.0017262 (PMC3043097; doi:10.1371/journal.pone.0017262)

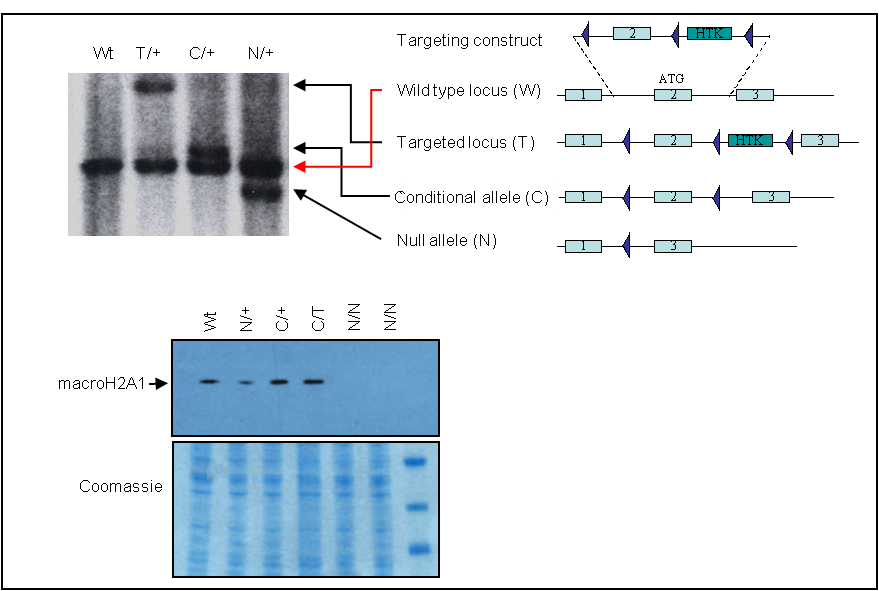

Supplement: Figure S1 — Generation of macroH2A1 V6.5 ES KO cells. A. The second exon of macroH2A1 contains the translation initiation codon. The next in-frame ATG is close to the end of the protein and several out-of–frame sites are found between the two. Thus, removal of exon 2 should completely prevent production of the protein. The targeting vector included a genomic fragment that covers exon 2 with flanking regions. LoxP sites (blue triangles) and the Hygro-TK (HTK) selection marker were inserted as shown. After transient exposure to Cre recombinase and selection with Ganciclovir two versions were obtained: in one version exon 2 was removed producing a null allele (N), in the second version only the HTK cassette was lost producing a conditional allele that can be looped out in a later stage to produce a null allele. On the left pane: Southern blot analysis (using a probe from macroH2A1 exon 3) demonstrating the correct targeting of ES cells carrying a targeted allele (T/+) a conditional allele (C/+) or a null allele (N/+). In C/+ cells the targeting procedure was repeated and after transient exposure to Cre recombinase, N/N cells were obtained. B. Western blot using the macroH2A1-NHR antibody confirms that knockout (N/N) cells do not express macroH2A1 in. Coomassie stain is used as loading control. (TIF) [file pone.0017262.s001.tif]
